# Early identification of esophageal squamous neoplasm by hyperspectral endoscopic imaging

**DOI:** 10.1038/s41598-018-32139-1

**Published:** 2018-09-14

**Authors:** I-Chen Wu, Hao-Yi Syu, Chun-Ping Jen, Ming-Yen Lu, Yi-Ting Chen, Ming-Tsang Wu, Chie-Tong Kuo, Yu-Yuan Tsai, Hsiang-Chen Wang

**Affiliations:** 1 Division of Gastroenterology, Department of Internal Medicine, Kaohsiung Medical University Hospital, Kaohsiung Medical University, No. 100 Shih-Chuan 1st Road, Kaohsiung, 80708 Taiwan; 2 Graduate Institute of Opto-Mechatronics, National Chung Cheng University, 168 University Rd., Min-Hsiung, Chia-Yi, 62102 Taiwan; 3 Department of Mechanical Engineering, National Chung Cheng University, 168 University Rd., Min-Hsiung, Chia-Yi, 62102 Taiwan; 4 Department of Materials Science and Engineering, National Tsing Hua University, 101, Sec. 2, Kuang-Fu Road, Hsinchu, 30013 Taiwan; 5 Department of Pathology, Kaohsiung Medical University Hospital, Kaohsiung Medical University, No. 100 Shih-Chuan 1st Road, Kaohsiung, 80708 Taiwan; 6 Department of Public Health, Graduate Institute of Clinical Medicine, Research Center for Environmental Medicine, Kaohsiung Medical University, No. 100 Shih-Chuan 1st Road, Kaohsiung, 80708 Taiwan; 7 Department of Physics, National Sun Yat-sen University, 70 Lienhai Rd., Kaohsiung, 80424 Taiwan; 8 Department of Gastroenterology, Kaohsiung Armed Forces General Hospital, 2, Zhongzheng 1st.Rd., Lingya District, Kaohsiung City, 80284 Taiwan

## Abstract

Esophageal squamous neoplasm presents a spectrum of different diatheses. A precise assessment for individualized treatment depends on the accuracy of the initial diagnosis. Detection relies on comprehensive and accurate white-light, iodine staining, and narrow-band imaging endoscopy. These methods have limitations in addition to its invasive nature and the potential risks related to the method. These limitations include difficulties in precise tumor delineation to enable complete resection, inflammation and malignancy differentiation, and stage determination. The resolution of these problems depends on the surgeon’s ability and experience with available technology for visualization and resection. We proposed a method for identifying early esophageal cancerous lesion by endoscopy and hyperspectral endoscopic imaging. Experimental result shows the characteristic spectrum of a normal esophagus, precancerous lesion, canceration, and intraepithelial papillary capillary loop can be identified through principal component score chart. The narrow-band imaging (NBI) image shows remarkable spectral characteristic distribution, and the sensitivity and specificity of the proposed method are higher than those of other methods by ~0.8 and ~0.88, respectively. The proposed method enables the accurate visualization of target organs, it may be useful to capsule endoscope and telemedicine, which requires highly precise images for diagnosis.

## Introduction

Esophageal cancer (ECA) largely differs from other malignant tumors in geographical distribution. This cancer type is more likely to occur in males than in females, and the difference between the maximum and minimum morbidity prevalence can reach ~100× in different countries. In ECA-vulnerable regions, including China and northern Iran, the predominant histological type is squamous cell carcinoma (SCC)^1–3^. Moreover, ECA ranks eighth among known cancer types worldwide in morbidity, yielding an annual increase of ~4.8 million cases, and sixth in mortality, accounting for 4.878% of the mortality caused by cancers^4^. The five-year survival rate of ECA can reach >90% in its early stage but is reduced to ~10% in its advanced stage^5^. Any evident symptom may not exist in the early stage of ECA until its advanced stage is reached, where dysphagia occurs owing to obvious obstruction caused by continuously enlarged tumor^6,7^.

Early local endoscopic detection and diagnosis are the key factors that reduce death rate; white-light endoscopy is a procedure that enables the early detection of ECA for medical treatment but not of lesions, which can be easily ignored, delaying treatment^8–12^. The application of chemical and optic theories to endoscopy led to the development of iodine staining endoscopy (Lugol chromoendoscopy) and narrow-band imaging (NBI) endoscopy, which highlight small esophageal cancerous lesions and improve the diagnostic rate of early cancer^13–16^. In iodine staining endoscopy, a stain is sprayed for the development effect. However, such spraying causes difficulty during evaluation because of uneven stain distribution. The stain may also cause chest discomfort as some patients are allergic to iodine. Meanwhile, certain subjectivity also exists in NBI analysis, and NBI is affected by several factors. For example, the view turns black under NBI in the presence of blood because light ray of 415 and 540 nm are absorbed by blood on the mucosal surface and not reflected, making the lesion unclear and difficult for clinical evaluation. NBI is the current mainstream technology for ECA detection because it is easy to operate and enables optical staining without stain spraying. White light can be randomly shifted to NBI mode through a single key; this feature is suitable to repeated observations; however, the main basis of detection is the changes in surface blood vessels, such as intraepithelial papillary capillary loop (IPCL)^11,17,18^, which are highly dependent on the subjective judgment of physicians. In this regard, we proposed a novel optical detection method, adopting hyperspectral image technology and principal component analysis (PCA) theory. The method allows the comparison among the results of a principal component score chart regarding the IPCL morphological changes of esophageal cancerous lesion in NBI endoscopic image and the characteristic spectrum of normal esophagus, precancerous lesion, and cancerated lesion in white light and iodine staining endoscopic images. The spectrum characteristic can assist physicians in rapid identification of early cancerous lesions in the esophagus.

## Results

### Endoscopic images

**Figure 1:**
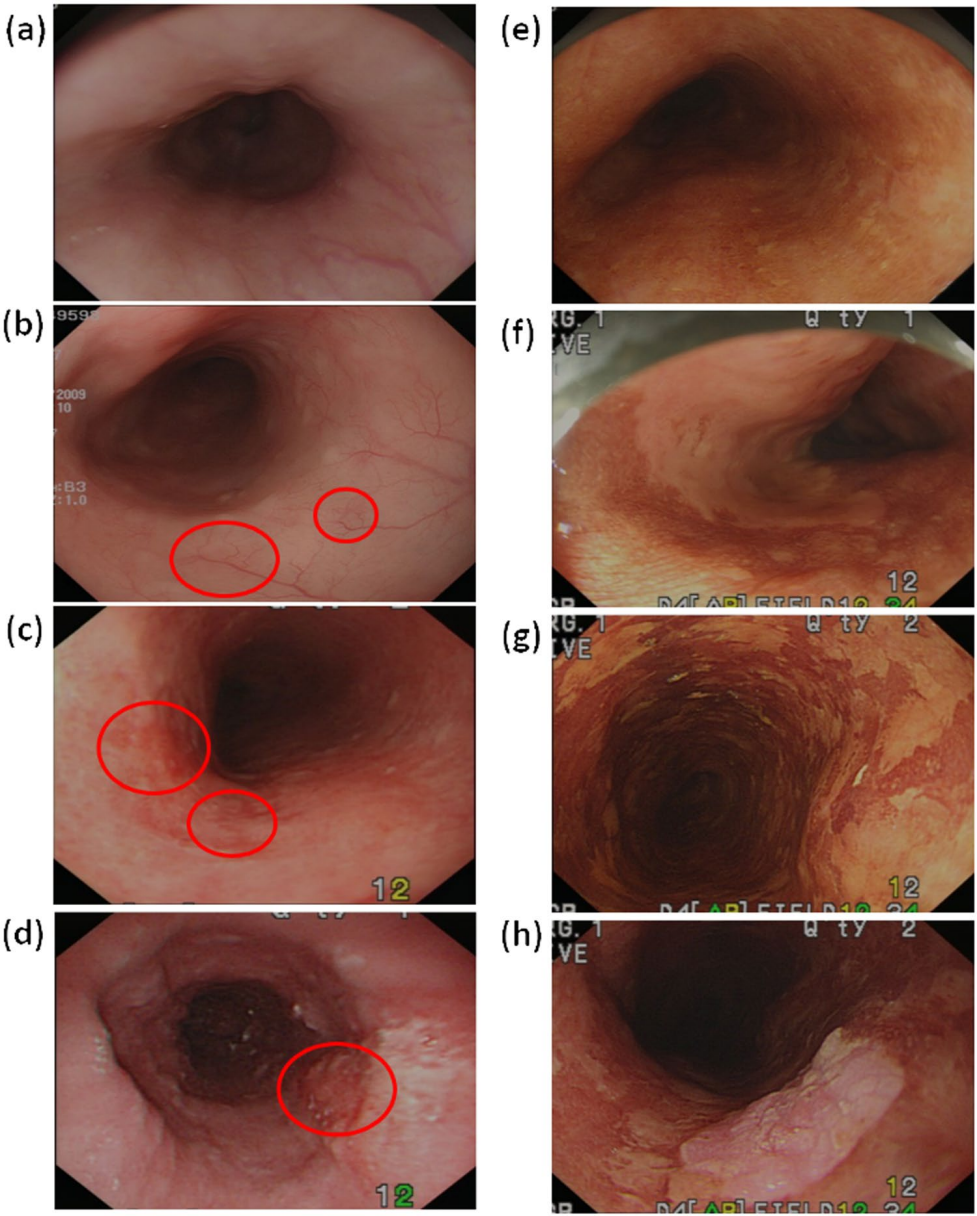
Images of white-light endoscopy: (**a**) normal, (**b**) low-grade dysplasia, (**c**) high-grade dysplasia, and (d) squamous cell carcinoma (SCC). Images of Lugol chromoendoscopy: (**e**) normal; (**f**) one large patch of Lugol-voiding lesion, which is suggestive of dysplasia; (**g**) multiple Lugol-voiding lesions (pathology: high-grade dysplasia); and (**h**) SCC.

Under white-light endoscopy, superficial ECA usually presents as an uneven and reddish lesion with unclear boundary and sometimes thin and white coating on the mucosal surface. The minute structure of esophagus mucosa cannot be clearly observed in conventional white-light endoscopic detection^19,20^, and diagnosis is made through biopsy and staining technology. Figure 1(a) shows the white-light endoscopic image of a normal esophagus, in which mucosal epithelium is semi-transparent, and the vascular network underneath the mucosa is clear and visible. Figure 1(b) presents the white-light endoscopic image of esophagus mucosa with dysplasia. The red frame shows mucosa incrassation and change in vascular structure. Figure 1(c) illustrates the white-light endoscopic image between dysplasia and ECA. When the mucosal epithelium is cancerated, the vascular network is invisible. As shown in the red frame, the mucosal color is changed by showing red area with unclear boundary. The mucosa is also slightly coarse and muddy. Figure 1(d) shows the white-light endoscopic image of ECA, in which the lesion is elevated and the vascular network is invisible. Figure 1(e–h) present the iodine staining endoscopic image of the esophagus. In the normal esophagus, a large amount of glycogen exists in the squamous epithelial cells of the mucosa. Glycogen exhibits strong affinity toward iodine solution and is thus stained brown [Fig. 1(e)]. Figure 1(f) illustrates a semi-circumferential patch sparing of iodine staining, which is suggestive of dysplasia. When a lesion occurs in the mucosa, cells with a considerable amount of glycogen will be reduced or disappear. Consequently, several areas without iodine staining will be observed. Figure 1(g) shows multiple blocky areas without iodine staining. Pathology confirmed the cells to exhibit high-grade dysplasia. Figure 1(h) presents ECA with a clear margin under Lugol chromoendoscopy. The adjacent flat areas without iodine staining are the surrounding dysplasia or superficial SCC. Iodine staining is the universal endoscopy staining. The intrinsic limitations of staining, such as uneven distribution of stain concentration and improper spraying method, results in uneven staining color of lesion areas, inaccurate positioning, or omission of lesion. According to the previous illustration, each staining method shows individual limitations, and double staining can be used for improvement.

### IPCL and NBI images

**Figure 2:**
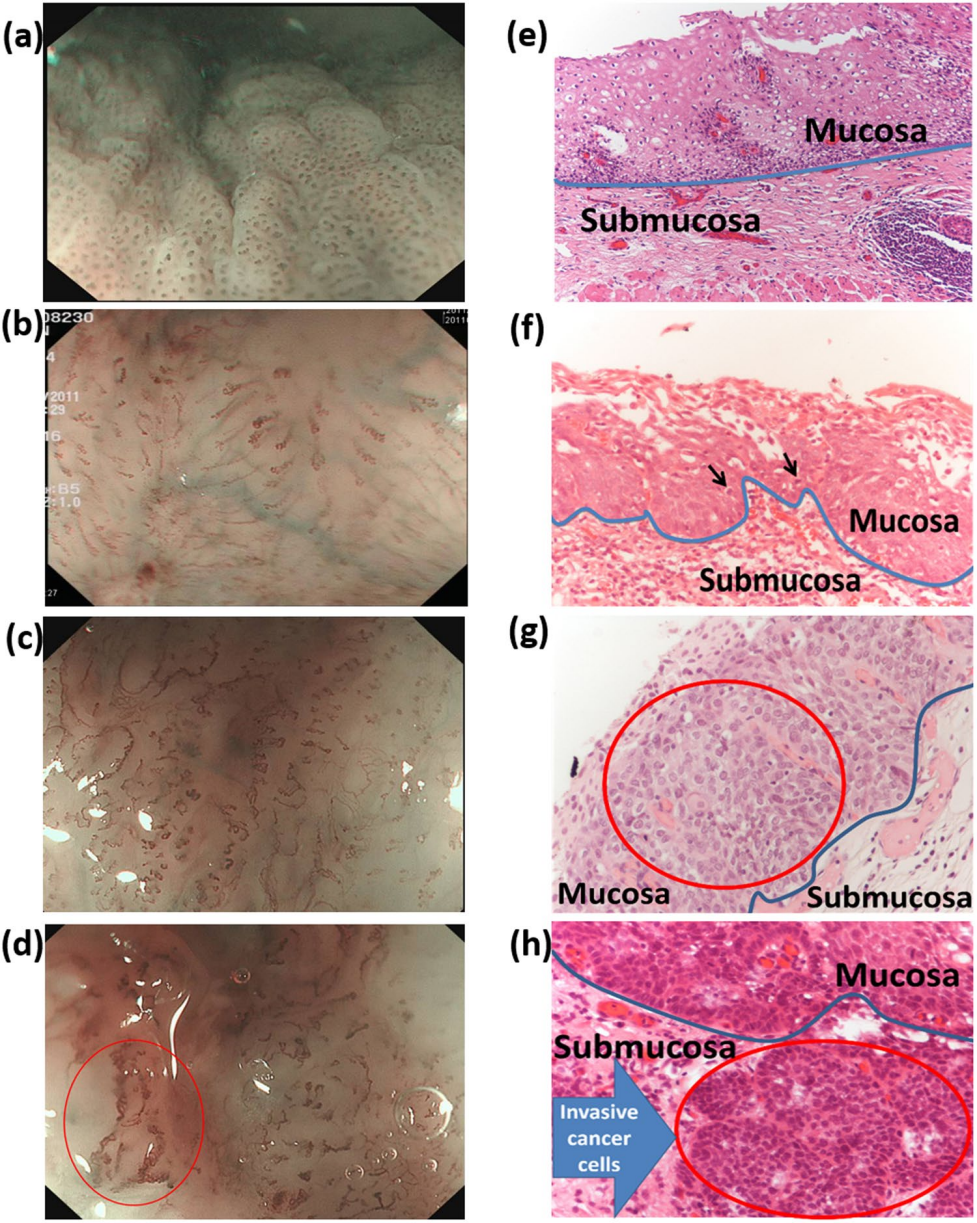
(**a**–**d**) Images of magnifying endoscopy with narrow band imaging: (**a**) intraepithelial papillary capillary loop (IPCL)-IV high-grade dysplasia, (**b**) IPCL-V1 high-grade dysplasia, (**c**) IPCL-V3 squamous cell carcinoma (SCC), and (**d**) IPCL-VN SCC. (**e**–**h**) Histopathological pictures (200×): (**e**) normal, (**f**) high-grade dysplasia, (**g**) high-grade dysplasia, and (**h**) SCC invading the submucosa (blue arrow).

IPCL is the annular capillary perpendicular to the subcutaneous smooth branches. Morphological changes in IPCL during esophageal canceration have been observed, such as dilation, distortion, and irregular change in caliber and shape—the four featured marks of cancer. These featured marks are useful in the assessment of atypia and invasive depth of cancer^8^. Under magnifying endoscopy, IPCL changes are divided into types I to V according to the Inoue classification method^10,11^. Type III and IV IPCL are commonly observed in low-grade and high-grade intraepithelial neoplasia, respectively. Type V IPCL can be divided into the following subtypes: type VI corresponds to carcinoma *in situ*; Type VII, VIII, VN represent further changes in tumor vessels, implying deeper mucosal or submucosal invasion^10,11^. Classification can also be made according to the invasive depth of tumor^11^. In mucosa cancer 1 (m1), the tumor is limited within the epithelium; in m2, in the lamina propria mucosae; in m3, in muscularis mucosae; in submucosa cancer 1 (sm 1), in the surface layer of submucosa; in sm 2, in the interlayer of submucosa; in sm 3, in the deep submucosal area^11^. Kumagai *et al*. classified the IPCL morphology under magnifying endoscopy through a combination of surgical specimen observation under stereoscopic microscopy and the corresponding pathological results. The correlation between IPCL pattern and histopathology reaches 83.3%^9^. Figure 2(a–d) depict the magnifying endoscopic image with NBI. Figure 2(a) is an IPCL-IV lesion with pathology-confirmed high-grade dysplasia, demonstrating slightly incrassated, dilated, and twisted IPCL and thus revealing a spiral form with irregular arrangement. Figure 2(b) presents IPCL-V1 with pathology-confirmed high-grade dysplasia (m1, in which the IPCL shows dilation, distortion, different diameters, and irregular shape). Figure 2(c) illustrates IPCL-V3 (SCC), in which IPCL exhibits remarkably damaged IPCL structure. Figure 2(d) reveals the IPCL-VN (SCC) with generation of bulky tumor vessels. Figure 2(e–h) present the pathological images of hematoxylin and eosin stain of the esophagus. In the esophageal tissue sections, the area containing nucleic acid is stained bluish violet, whereas the area containing protein is stained pink; the blue line separates the upper and bottom layers of the mucosa^21–23^. Figure 2(e) shows the image of normal esophagus tissue section, in which the tissue structure of squamous epithelium is well-preserved; in the upper mucosal layer, nuclear/cytoplasmic (N/C) ratio is reduced as the cytoplasm is increased from the substrate to the superficial layer. Figure 2(f) presents the image of high-grade dysplasia, in which some cancer cells with increased N/C ratio and hyperchromasia are present (black arrows). Figure 2(g) illustrates the image of high-grade dysplasia; the numerous red globoids in the red circle are cancer cells that have not infiltrated the submucosa (blue line). The corresponding tumor infiltration depth is m1. Figure 2(h) shows the image of SCC, in which cancer cells have invaded the submucosa (red circle) and the corresponding tumor infiltration depths are m3 and sm1.

**Figure 3:**
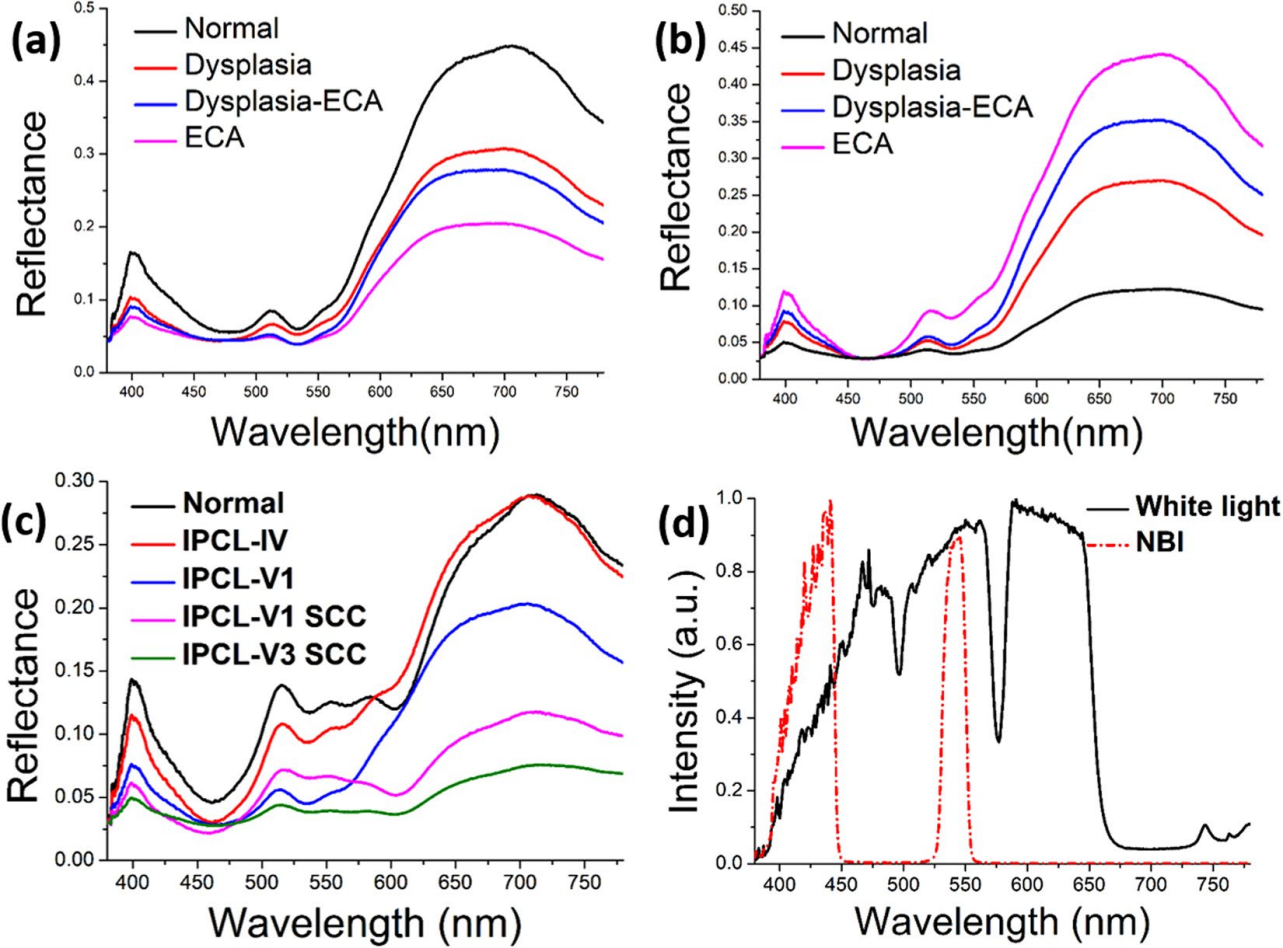
Average reflection spectra of normal, dysplasia, dysplasia–ECA, and ECA esophageal mucosa: (**a**) white-light endoscopy; (**b**) Lugol’s chromoendoscopy; (**c**) average reflection spectra of IPCL-IV severe dysplasia, IPCL-V1 severe dysplasia, IPCL-V1 SCC, and IPCL-V3 SCC esophageal mucosa by NBI; and (**d**) spectra of white light and NBI.

The HSEI calculation results of the average spectra of normal esophageal mucosa, dysplasia, dysplasia-ECA, and ECA by white-light endoscopy and Lugol chromoendoscopy are shown in Fig. 3(a,b), respectively. Figure 3(c) shows the HSEI calculation results of IPCL-IV high-grade dysplasia, IPCL-V1 high-grade dysplasia, IPCL-V1 SCC, and IPCL-V3 SCC by NBI. Each average spectrum was calculated from the amount of data at 2500 points (50 × 50 pixels for each patient) for white-light endoscopy and Lugol chromoendoscopy images. Figure 3(a) shows that the difference in reflection spectrum depends on the degree of lesion (normal, dysplasia, dysplasia-ECA, and ECA) with decreasing tendency of spectrum reflectivity. In general, the normal esophageal mucosa is whiter than the gastric mucosa. When precancerous lesion and cancerous focus occur, uneven color can be caused by sunken or ridged lesions on the esophageal mucosal surface. Mucosal lesions show a darker red color than the surrounding mucosa, which causes low spectrum reflectivity for severe lesions. The corresponding spectrum reflectivity of blue and green ray wave bands is evidently lower than that of a red ray wave band. The spectrum reflectivity at 530 nm wavelength is reduced because the angiogenesis in mucosal tissues caused by ECA supplies increased nutrition and oxygen for cancer cells, and the absorption rate of increased hemoglobin is stronger than those of blue and green rays^24^. Additionally, wave crests are observed at wavelengths of ~410 and ~520 nm. Further study on pertinent literature is required for the identification of the underlying causes of the result. Meanwhile, as shown in Fig. 3(b), the differences among the reflected spectra of the iodine staining endoscopic images depend on normal, precancerous lesion, and cancer, with the tendency to gradually increase in spectrum reflectivity. This phenomenon is observed because the normal esophageal mucosa is part of the squamous epithelial cells, and reaction occurs between iodine solution and glycogens in cells to stain the mucosa to brown. When cancer cells invade the epithelium, glycogen is reduced or disappears. Consequently, the iodine solution cannot stain the lesion. Therefore, the unstained area on the mucosa is possibly a cancerous lesion, and the severity of the lesion in the area depends on the white color compared with the surrounding mucosa. The NBI endoscopic image adopts a direct automatic circle of IPCL morphology so that the sampling pixel remains large. However, a total of 1000 coordinate points will be selected during the experiment. These points correspond to 1000 sets of reflected spectrum. The reflection spectrum tendency of NBI endoscopic image is shown in Fig. 3(c). The difference is that IPCL shows a dilated, twisted, and irregular morphology according to the canceration degree of lesions, thereby gradually declining the tendency of spectrum reflectivity. The overall trend below the 580 nm is consistent with the white-light endoscopy results. But the reflectance spectrum of IPCL-IV have higher values than normal condition between 580–700 nm. NBI endoscopy filters the red ray components in white light to leave narrow-band blue and green rays, and its imaging involves complicated image processing of color conversion and color reproduction^25,26^. Thus, we can only identify the general difference of spectrum reflectivity but cannot explain the reason for such tendency. The limitation of the present study is that both hospitals could not provide NBI endoscopic images of IPCL-II, and IPCL-III morphology. We can only observe the difference through spectrum tendency according to high-grade dysplasia, SCC, and normal. However, the difference in IPCL morphology of canceration degree can be observed from the result in Fig. 3(c). The average reflection spectrum of the three endoscopic images shows no change with the influence of light source of endoscopy. The light source spectrum of endoscopy is shown in Fig. 3(d).

## Discussion

The present result indicates two major factors influencing the reflection spectrum of endoscopic image. First, the uneven surface of esophagus mucosa within the wavelength of 570–780 nm reduces spectrum reflectivity. Second, increased hemoglobin caused by ECA angiogenesis within the wavelength of 380–570 nm increases the absorption rates of blue and green rays. By contrast, the spectrum reflectivity is reduced. Furthermore, the absorption rate peaks at 530 nm wavelength, and wave crests are formed at 410 and 520 nm wavelengths. The reason for these observations remains unclear. The exact influencing factor has been described in literature. The focus of this study is the variation in reflection spectrum. We observed evident difference between the reflection spectra of iodine staining endoscopic and white-light endoscopic images. Controversy exists in the reflection spectrum of NBI endoscopic image owing to the involvement of complicated image processing technology, such as color conversion and color reproduction, which are lacking in this study. The characteristic spectra of white light, iodine staining, and NBI endoscopic images can be clearly identified by using PCA. The characteristic spectrum of NBI endoscopic image shows lesser fuzzy region problem than those of the two other images. The tendency is also evident. Therefore, variation in reflection spectrum can be determined from an iodine staining endoscopic image. Meanwhile, samples for the identification of tendency in characteristic spectrum through principal component score chart can be obtained from NBI endoscopic images.

**Figure 4:**
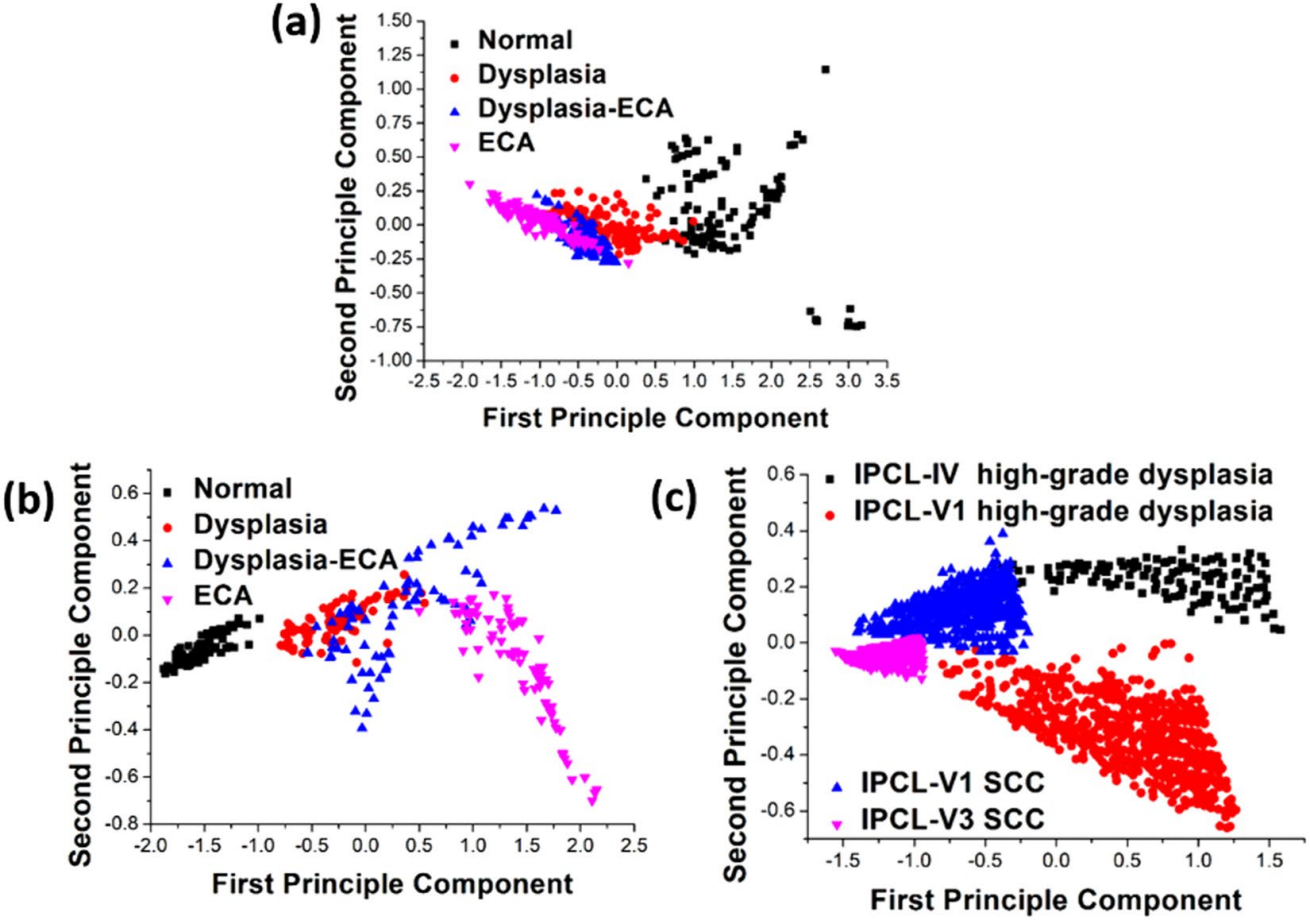
Principal component distribution diagram of the four kinds of early cancerous changes: (**a**) white-light endoscopy, (**b**) Lugol chromoendoscopy, and (**c**) NBI.

**Figure 5:**
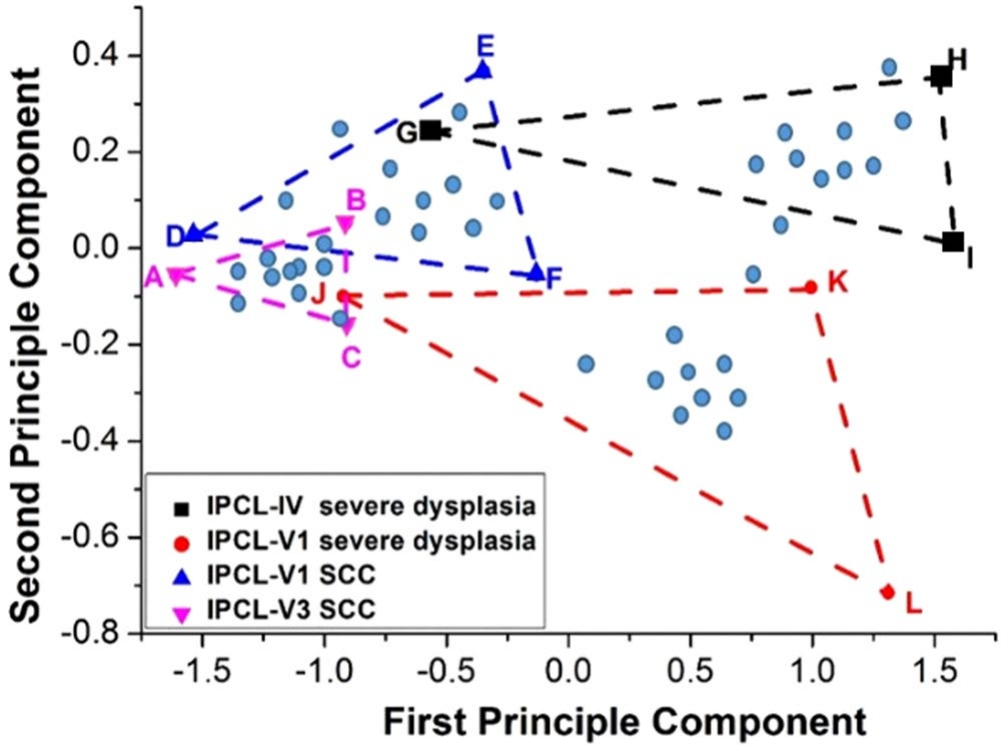
Defining triangle areas in the PCA FS.

The overlap area of NBI image through HSEI analysis is small and restraint within a fixed scope. Thus, a method for the identification of esophageal canceration degree is established. In Fig. 4(c), three points in the peripheral area are selected according to different cancerous staging distributions. The triangular scope of the four staging types is outlined, as shown in Fig. 5. The average principle component score of the stimulating spectra of the image in the NBI endoscopy of a patient is determined whether it is located in the triangular areas to evaluate the cancerous stages occurring in the patients. The detection method of the triangle areas is used. As shown in Fig. 5, the image spectra obtained in the endoscopy by stimulating and analyzing the HSEI system are applied in the principle component score diagram. The obtained average stimulating spectra are classified, and the triangular area is defined as the maximum area of the triangle in the principle component score diagram generated by the PCA of the aforementioned triangle area. For example, one point at the left hand side in the x axis is selected as a reference point, and two other points are randomly selected. The area of these three points is calculated by a computer, and two other points are subsequently selected to calculate another triangular area. Afterward, the two triangular areas are compared for the determination of the large triangular area. In accordance with the comparison method of the triangular area and the computer calculation, the triangle with maximum area can be quickly determined in the principle component score diagram. The three coordinates of the three points for the triangle with maximum area may also be found. When the three coordinates for the triangle area are obtained, the coordinates of vectors AB and AC can also be found. For example, when the three coordinates of the triangular area are A (a1, a2), B (b1, b2), and C (c1, c2), vector AB = (b1-a1, b2-a2), vector AC = (c1-a1, c2-a2), and the area of ΔABC is as follows:

1 $${\rm{\Delta }}\mathrm{ABC}=\frac{1}{2}\Vert \begin{array}{cc}b1-a1 & c1-a1\\ b2-a2 & c2-a2\end{array}\Vert $$

In accordance with the ΔABC area, when one X point is given, and the coordinate is X(s, t), the ΔXAB, ΔXBC, and ΔXAC areas can be calculated. When the X point is located outside the ΔABC, the following condition will be satisfied:

2 $${\rm{\Delta }}\mathrm{XAB}+{\rm{\Delta }}\mathrm{XBC}+{\rm{\Delta }}\mathrm{XAC} > {\rm{\Delta }}\mathrm{ABC}$$

When X point is located at the edge of or outside of the ΔABC, the following condition will be satisfied:

3 $${\rm{\Delta }}\mathrm{XAB}+{\rm{\Delta }}\mathrm{XBC}+{\rm{\Delta }}\mathrm{XAC}={\rm{\Delta }}\mathrm{ABC}$$

According to the determination method of the triangular area, the location of the principle component score of the stimulating spectra of the image in the endoscope of a patient within one of the aforementioned and defined triangular areas, such as the area of the IPCL-IV high-grade dysplasia, IPCL-V1 high-grade dysplasia, IPCL-V1 SCC, and IPCL-V3 SCC, may be known. Finally, when the principle component score of the second stimulating spectrum is located within one of the triangular areas, the second pathology image is confirmed as one cancerous lesion image, and the four different cancerous lesions are detected. The average principle component score of a patient is set as a testing point. When the testing point is located within the triangular area, the lesion image of the testing point belongs to the stage of the cancerous lesion. For example, the ECA shows 1–4 stages: (1) IPCL-V3 SCC. U is the testing point. When ΔUDE + ΔUEF + ΔUDF = ΔDEF is satisfied, the patient belongs to the cancerous lesion stage. (2) IPCL-V1 SCC. When ΔUDE + ΔUEF + ΔUDF = ΔDEF is satisfied, the patient belongs to this cancerous lesion stage. (3) IPCL-V1 V1 high-grade dysplasia. When ΔUDE + ΔUEF + ΔUDF = ΔDEF is satisfied, the patient belongs to this cancerous lesion stage. (4) IPCL-IV high-grade dysplasia. When ΔUDE + ΔUEF + ΔUDF = ΔDEF is satisfied, the patient belongs to this cancerous lesion stage. (5) When the testing point of U does not satisfy the aforementioned condition, U cannot be detected. According to the cancerous lesion identification method by the HSEI, the cancerous lesion image is digitized. Additionally, the PCA is used for the quick evaluation of the probability of cancer occurrence at each stage. We included additional 60 patients with esophageal SCC in the past medical database to determine the diagnosis sensitivity and specificity of this method. The average PCA values of these patients are shown in Fig. 5 as blue points. Image analysis and data processing are shown in Supporting Information S4. The diagnosis sensitivity of IPCL-IV, IPCL-V1, IPCL-V1 SCC, and IPCL-V3 SCC is 0.73, 0.8, 0.66, and 0.8, respectively. The diagnosis specificity of four ECA stages is 0.97, 1.0, 0.93, and 0.88, respectively. These sensitivity and specificity values are not significant in this study due to the insufficient number of patients. However, we still believe that the proposed method can effectively and rapidly help patients to obtain early-stage treatment. In conclusion, according to the present results, the identification of early cancerous esophageal lesions by hyperspectral image technology enables rapid diagnosis. To identify ESCC earlier, the physician can select the suspicious area in the endoscopic esophageal image. The spectral information of each pixel in the selection area will be calculated. The disease degree of selection area can be determined through the HSEI database. The physician can identify ESCC earlier through the color markers of selection area in the monitor.

## Methods

### Sample preparation

All images of the patients and the pathological sections in this study were provided by the Division of Gastroenterology of Kaohsiung Medical University Hospital. We included 220 patients, including 60 normal, 56 dysplasia, 49 intermediate phase between dysplasia and ECA, and 55 ECA cases, to build the hyperspectral endoscopic imaging (HSEI) database.

### Ethical Statement

The study protocol and informed consent forms were approved by the Institutional Review Board of the Kaohsiung Medical University Hospital (IRB Number: KMUHIRB-E(I)-20170271). Informed consent was obtained from all participants. All methods were performed in accordance with the relevant guidelines and regulations. Investigate by the ethics committee of the Kaohsiung Medical University Hospital, the experiment design and experiment process is not involved ethical experiments.

### HSEI

Many researchers applied the hyperspectral image technology in various medical fields, particularly in the early detection of oral cancer^27^; detection of oral lesion caused by enterovirus^28,29^, skin lymph cancer^30,31^, and blood oxygen concentration of skin^32,33^; identification of fungal taxonomy^34^; and bone marrow cell detection^35^. The first type of hyperspectral image system adopts a single-point spectrometer supported with a 2D scanning system^36^. Although this system can achieve an optimal simulated spectrum and space resolution, it requires a considerable amount of time for data reading. The second hyperspectral image system adopts a camera supported with an LC-coordinated filter and a microscope, which can be applied to detect bone marrow cells^37^. This method can classify the components in bone marrow cells, but the effect of LC-coordinated filter limits the reading speed of spectrum data. The third type of hyperspectral image system adopts a hyperspectral camera for spectrum and image analysis, and this system has been successfully applied to cosmetology and skin detection^38^. Such system also possesses considerably high image resolution, but handling large data requires a high cost. In the present study, we developed a hyperspectral image system composed of a single-lens reflex camera (Olympus, E520) and a high-definition spectrometer (Ocean Optics, QE65000). We obtained relatively good results from applications, including a study on color gain technology for skin lymph cancer^30,31^. We obtained the spectrum of each pixel in the images of patients with skin disease through hyperspectral image technology and identified different skin diseases through PCA and principal component score chart. We designed a spectrum gain technology to effectively enhance the color shading by >10% between normal and lesion area in images of patients with skin lymph cancer. Multispectral imaging microscopy (MSI) was used for the identification of the cellular staging of bladder cancer. Hyperspectral image, color reproduction technology, and PCA were used for the classification of normal bladder cells (E7), stage II bladder cancer (TSGH-8301), stage III bladder cancer (J82), and stage IV bladder cancer (TCC-SUP) into three categories by means of an elliptic equation, that is, normal (stages II and III) and stage IV^38,39^. The two applications effectively promote the diagnostic efficiency of doctors and help patients obtain early treatment through hyperspectral image technology.

**Figure 6:**
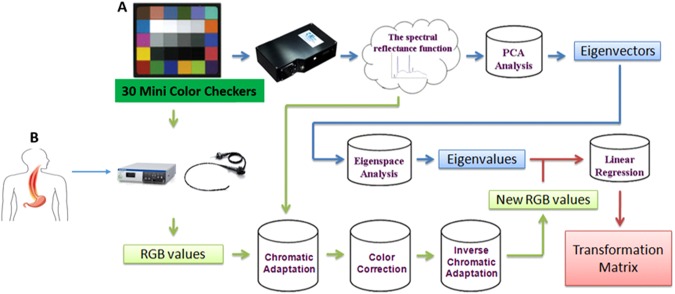
Schematic diagram of the proposed method used in estimating the spectral reflectance of each pixel in an image using endoscopy.

The estimated spectral processes of the HSEI data are illustrated in Fig. 6. HSEI was used to obtain the spectrum of each image element of an endoscopic imaging. The HSEI was divided into three parts: the PCA for spectral data reduction, the calculation of transformation matrix to determine the relationship between the endoscopy (Olympus CV-290) and the spectrophotometer (Ocean Optics, QE65000), and the spectral reproduction of images. The experimental results and the calculations are reported in refs^27–31^. For an accurate estimation of the spectra, the process of addressing the red, green, and blue (RGB) values of each image element after capture was corrected. Color correction was implemented to match the color performance of the camera with that of the spectrophotometer. According to the spectrophotometric data, the International Commission on Illumination/Commission Internationale de L'éclairage XYZ tristimulus values calculated and established the corresponding RGB values from the spectra of 30-color checkers (X-Rite, Mini Color Checkers) as the standard values. Images under similar lighting conditions were captured using endoscopy, and the RGB values of each image element were retrieved by a computer program. Finally, the color relationship between the two devices was determined by separately performing third-order polynomial regression for the RGB components. The output format of commercial endoscopy was sRGB (RAW image files), in which the reference white, which is different from the artificial lights used to measure the spectra of 30-color checkers, was provided by the xenon lamp of an endoscopy. Consequently, chromatic adaptation transformation was performed prior to the third-order polynomial regression. The detailed explanation of HSEI calculated processes are shown in Supporting Information S1.

### Automatic circling and pixel coordinates recording algorithm

**Figure 7:**
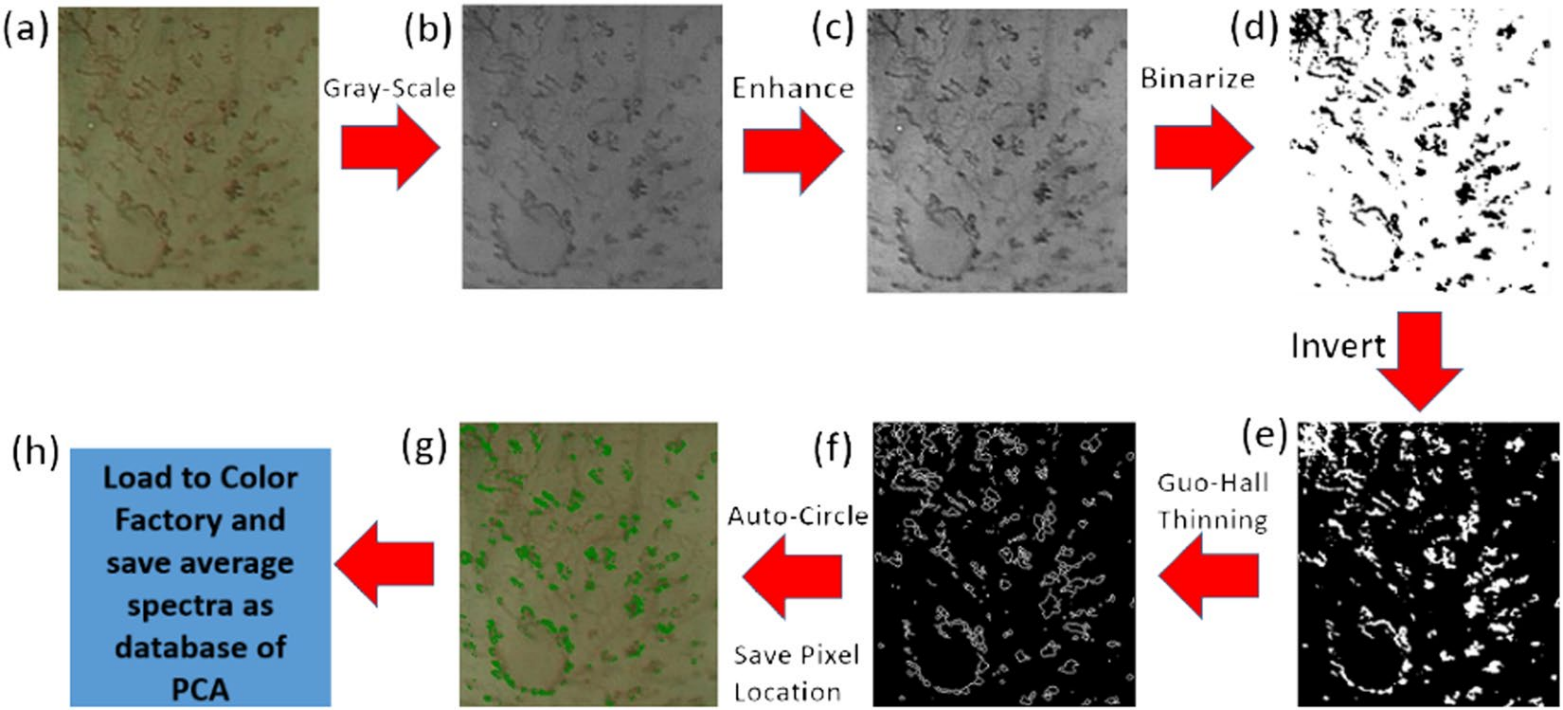
Flowchart of automatic circling and pixel coordinate recording algorithm: (**a**) endoscopic esophageal image, (**b**) grayscale result of (**a**) image, (**c**) contrast enhancement of (**b**) image, (**d**) binarization result of (**c**) image, (**e**) invert result of (**d**) image, (**f**) Guo–Hall thinning result of (**e**) image, and (**g**) automatic circle and program-distinguished image of (**f**), and (**h**) saved average spectra as database of principal component.

We adopted the automatic image selection method reduce analysis time. Figure 7 shows the flow diagram of image automatic selection method. First, the endoscopic image is converted into gray scale through rgb2gray(x) order of Matlab. The enhanced image is compared by formula S15 in supporting information. Binarization is conducted on the enhanced image through the im2bw order of Matlab. A program written with Visual Basic is used for the recording of the IPCL pixel coordinates of the NBI endoscopic image after binarization. The location of IPCL in the image is then selected according to the threads surrounding the IPCL through a thinning process. The recorded pixel coordinates is exported to Notepad. NBI endoscopic image is read through the interface of Color Factory, and the pixel coordinates in Notepad are read simultaneously. Finally, the spectrum information of each pixel in the image is obtained through hyperspectral image technology. The detailed explanation of this algorithm is shown in Supporting Informations S2 and S3.

4 $$\frac{pixel(x,y)-\,\min [pixel(x,y)]}{\max [pixel(x,y)]-\,\min [pixel(x,y)]}\times 255$$

### PCA

PCA is a common method in multivariate statistics^40^. This technique has been applied to color technology since 1960. PCA involves the determination of a subspace that is less than the original variable, maintaining data change in a multivariable data set and projecting the original data into the subspace for analysis^41^. A detailed explanation of PCA-calculated processes is discussed in ref.^38^. The characteristic spectra of white light, iodine staining, and NBI endoscopic images are usually obtained through PCA. The relation graph of first and second principal components is calculated. Figure 4 presents the principal component score chart. Figure 4(a) presents the characteristic spectrum of white light endoscopic image, in which the drop point of ECA locates between −1.90 and 0.25 of the first principal component (FPC) and between −0.35 and 0.35 of the second principal component (SPC). Dysplasia-ECA ranges from −0.90 < FPC < 0.05 to −0.30 < SPC < 0.30. Dysplasia is between −0.75 < FPC < 1.00 and −0.20 < SPC < 0.35, and the normal range is between 0.30 < FPC < 3.25 and −0.75 < SPC < 1.25. Nonetheless, an overlap exists among dysplasia, dysplasia-ECA, and ECA. Such overlap is regarded as a fuzzy region. The characteristic spectrum of the endoscopic image of normal esophagus mucosa shows diversion tendency, but the difference from normal esophagus mucosa to canceration is observed from right to left. The characteristic spectrum of iodine staining endoscopic image is shown in Fig. 4(b). The normal range is between −1.90 < FPC < −0.90 and −0.19 < SPC < 0.10. The scopes of dysplasia, dysplasia-ECA, and ECA are between −0.80 < FPC < 0.50 and −0.16 < SPC < 0.28, −0.40 < FPC < 1.70 and −0.42 < SPC < 0.56, and 0.00 < FPC < 2.20 and −0.74 < SPC < 0.18, respectively. However, similar to what is observed in the NBI endoscopic image, a fuzzy region exists among dysplasia, dysplasia-ECA, and ECA. A diversion tendency is also observed in the endoscopic images of dysplasia-ECA and ECA of esophageal mucosa. Furthermore, a difference in the characteristic spectrum can be generally observed. The tendency is from left to right according to normal esophagus mucosa to canceration. The characteristic spectrum of NBI endoscopic image is shown in Fig. 4(c), in which 20 patients for each of the four types of canceration degree are shown. Four IPCL locations are selected. Each location shows the calculation result of 2500 pixels. IPCL-V3 SCC, IPCL-V1 SCC, IPCL-V1 high-grade dysplasia, and IPCL-IV high-grade dysplasia lie within −1.70 < FPC < −0.90 and −0.15 < SPC < 0.02, −1.40 < FPC < −0.30 and −0.02 < SPC < 0.40, −0.75 < FPC < 1.25 and −0.68 < SPC < −0.01, and −0.60 < FPC < 1.60 and 0.05 < SPC < 0.31. Notably, the fuzzy region formed in the four spectrum signatures is small, and the characteristic spectrum of IPCL morphology shows convergence tendency with increasing canceration degree in the esophagus.

## Electronic supplementary material

Supplementary Information
