# Supplementary material for: Early identification of esophageal squamous neoplasm by hyperspectral endoscopic imaging
Source: Sci Rep. 2018 Sep 14;8:13797. doi: 10.1038/s41598-018-32139-1 (PMC6138669; doi:10.1038/s41598-018-32139-1)
Supplement: Supplementary file 1 — Supplementary Information [file 41598_2018_32139_MOESM1_ESM.pdf]

Early identification of esophageal squamous neoplasm by hyperspectral endoscopic imaging

I-Chen Wu[1], Hao-Yi Syu[2], Chun-Ping Jen[3], Ming-Yen Lu[4], Yi-Ting Chen[5], Ming-Tsang Wu[6], Chie-Tong Kuo[7], Yu-Yuan Tsai[8,‡],and Hsiang-Chen Wang[2,*]

[1] Division of Gastroenterology, Department of Internal Medicine, Kaohsiung Medical University Hospital, Kaohsiung Medical University, No. 100 Shih-Chuan 1st Road, Kaohsiung 80708, Taiwan.

[2] Graduate Institute of Opto-Mechatronics, National Chung Cheng University, 168 University Rd., Min-Hsiung, Chia-Yi 62102, Taiwan.

[3] Department of Mechanical Engineering, National Chung Cheng University, 168 University Rd., Min-Hsiung, Chia-Yi 62102, Taiwan.

[4] Department of Materials Science and Engineering, National Tsing Hua University, 101, Sec. 2, Kuang-Fu Road, Hsinchu 30013, Taiwan.

[5] Department of Pathology, Kaohsiung Medical University Hospital, Kaohsiung Medical University, No. 100 Shih-Chuan 1st Road, Kaohsiung 80708, Taiwan.

[6] Department of Public Health, Graduate Institute of Clinical Medicine, Research Center for Environmental Medicine, Kaohsiung Medical University, No. 100 Shih-Chuan 1st Road, Kaohsiung 80708, Taiwan.

[7] Department of Physics, National Sun Yat-sen University, 70 Lienhai Rd., Kaohsiung 80424, Taiwan.

[8] Department of Gastroenterology, Kaohsiung Armed Forces General Hospital, 2, Zhongzheng 1st.Rd., Lingya District, Kaohsiung City 80284, Taiwan

S1. Hyperspectral Endoscopic Imaging (HSEI) calculated processes

The estimated spectral processes of the HSEI data are illustrated in Fig. 3. For the algorithm building as step A, the spectra of the 30 Macbeth color checkers are measured by a spectrophotometer (Ocean Optics QE65000) under the illumination of a particular uniform artificial light, and the reflection spectrum of each color checker in the visible light region (380–780 nm) is obtained. These spectra are arrayed as a matrix, $[D]_{401x30}$. The rows and columns indicate the intensities of the wavelengths at 1 nm intervals and the numbers of the color checkers, respectively. By determining the eigen-system and applying the PCA, we selected six eigen vectors that have substantial contribution as the bases of spectral estimation. The eigen vectors are arrayed as matrix $[E]_{6x401}$. The corresponding eigen values of these six eigen vectors $[\alpha]_{6x30}$ can be determined as follows:

$$[\alpha]^T = [D]^T pinv[E] \tag{S1}$$

where pinv denotes the pseudoinverse of the matrix. The color checkers are simultaneously captured by an endoscope (Olympus CV-290) under the same illumination condition, and the output format is sRGB (JPEG image files) [21]. The red, green, and blue values from 0 to 255 of each color checker image are obtained using computer programs and are then plotted on a scale of R_{srgb}, G_{srgb}, and B_{srgb} from 0 to 1. These RGB values can be transferred into CIE XYZ tristimulus values by the following formula [21] :

$$\begin{bmatrix} X \\ Y \\ Z \end{bmatrix} = [T] \begin{bmatrix} f(R_{srgb}) \\ f(G_{srgb}) \\ f(B_{srgb}) \end{bmatrix} \tag{S2}$$

where

$$[T] = \begin{bmatrix} 0.4124 & 0.3576 & 0.1805 \\ 0.2126 & 0.7152 & 0.0722 \\ 0.0193 & 0.1192 & 0.9505 \end{bmatrix} \tag{S3}$$

$$f(n)=\begin{cases}(\frac{n+0.055}{1.055})^{2.4} & , n>0.04045 \\ (\frac{n}{12.92}) & , otherwise\end{cases} \quad \text{(S4)}$$

These RGB values are corrected for chromatic adaptation by applying CMCCAT2000 because of the reference white of the sRGB color space illuminated by a CIE standard light source D65, which is different from the artificial light used for measuring the spectra of the color checkers [21]. Color correction of the endoscopy is also required, considering the accuracy of spectral estimation. The reflection spectra measured by the spectrophotometer are transferred into CIE XYZ tristimulus values by using Eqs. S5–S8. In these calculations, $S(\lambda)$ is the relative spectral power distribution of the artificial light, $R(\lambda)$ is the spectral reflectance of the respective color checker, and $\bar{x}(\lambda)$, $\bar{y}(\lambda)$, and $\bar{z}(\lambda)$ are the color matching functions.

$$X=k\int_{380nm}^{780nm}S(\lambda)R(\lambda)\bar{x}(\lambda)d\lambda \quad \text{(S5)}$$

$$Y=k\int_{380nm}^{780nm}S(\lambda)R(\lambda)\bar{y}(\lambda)d\lambda \quad \text{(S6)}$$

$$Z=k\int_{380nm}^{780nm}S(\lambda)R(\lambda)\bar{z}(\lambda)d\lambda \quad \text{(S7)}$$

where

$$k=100/\int_{380nm}^{780nm}S(\lambda)\bar{y}(\lambda)d\lambda \quad \text{(S8)}$$

After the chromatic adaptation transformation, the RGB values corresponding to the new XYZ values are calculated through the inverse procedures of Eqs. S2–S4 and then set as standard matrix $[A]$. The color relationship between the spectrophotometer and the endoscopy is found by using the third-order polynomial regression for the red, green, and blue components separately. The regression matrix $[C]$ is determined as follows:

$$[C]=[A]pinv[F] \tag{S9}$$

where

$$[F]=\left[1,R,G,B,RG,GB,BR,R^2,G^2,B^2,RGB,R^3,G^3,B^3,RG^2,RB^2,GR^2,GB^2,BR^2,BG^2\right]^T \tag{S10}$$

"R", "G", and "B" are the respective RGB values of the color checkers captured by the endoscopy. The corrected RGB values are obtained from Eq. S11, where [K] presents the RGB values captured from any image that is expanded into a format, such as the original matrix [*F*] and arrayed as a matrix $[\beta]$.

$$[\text{Corrected RGB}]=[\text{C}][\text{K}] \tag{S11}$$

Finally, a transform matrix $[M]$ between the spectrophotometer and the endoscopy is obtained as follows:

$$[M]=[\alpha]pinv[\beta] \tag{S12}$$

For each pixel in any image captured by the endoscopy (step B in Fig. 3), the RGB values multiplied by the regression matrix $[C]$ and the corresponding XYZ values are calculated by using Eqs. S2–S4. The estimated spectra of one endoscopy image in the visible light region (380–780 nm) are obtained by using the following formula:

$$[Spectra]_{380-780nm}=[E][M]\begin{bmatrix}X\\Y\\Z\end{bmatrix} \tag{S13}$$

Esophageal cancer presents a spectrum of different diatheses. A precise assessment for individualized treatment depends on the accuracy of the initial diagnosis. Detection relies on comprehensive and accurate white-light cystoscopy. In addition to its invasive nature and the potential risks related with the method, white-light cystoscopy exhibits limitations, including difficulties in flat lesion detection, precise

tumor delineation to enable complete resection, inflammation and malignancy differentiation, and grade and stage determination. The resolution of these problems depends on the surgeon's ability and experience with available technology for visualization and resection. In this study, we used hyperspectral imaging technology combined with endoscopy to analyze ECCs at various stages. The lesion's spectra at different cancer stages change the lesion's composition.

S2. Automatic circling and pixel coordinate recording algorithm

We used the flow chart in Fig. 5 as an introduction to encircle and select the lesion's part automatically. The chart is mainly divided into four parts: image capture (step 1), image preprocessing (steps 2–5), lesion's part judgment (step 6), and circle result (step 7).

Step 1: Image capture

Figure 5(a) refers to the endoscopic esophageal image captured by endoscopy (Olympus CV-290).

Step 2: Gray scale

The grayscale aims to simplify the image data for other related image processing techniques. These images are RGB color images. The RGB values of each pixel with 24-bit code are 8 bits each for red, green, and blue. The value ranges from 0 to 255. The grayscale image only shows 8-bit strength from 0 (black) to 255 (white). We transmitted

RGB values into gray using Eq. S14, and the result is shown in Fig. 5(b).

$$\text{Gray}=\text{R}\times0.299+\text{G}\times0.587+\text{B}\times0.114 \quad \text{(S14)}$$

Step 3: Contrast and enhancement

Grayscale distribution refers to the chart marking the occurrence of each grayscale in the image. In this chart, grayscale distribution is centered at 100–200. This condition indicates the closeness of the colors. The lesions and normal tissues are difficult to distinguish. Therefore, contrast enhancement is necessary. We adopted Eq. S15, the histogram stretching equation, to convert the image pixel with concentrated original distribution into a new pixel in 0–255 of the distribution range. MaxPixel and MinPixel refer to the maximum and minimum gray pixels in the original image, respectively. Results following the contrast enhancement performed in Fig. 5(b) are shown in Fig. 5(c).

$$\text{NewPixel}(i,j) = 0 + \frac{\text{Pixel(i,j)}-\text{MinPixel}}{\text{MaxPixel}-\text{MinPixel}} \times 255 \quad \text{(S15)}$$

Step 4: Binarization

Narrow-band imaging (NBI) is used to capture images in this study. Light is centered on the lesion’s sample for easy observation. Fig. 5(c) reveals that a selected part is

brighter than the other part after contrast enhancement. We filtered unwanted parts to determine the most suitable binarization threshold. The desirable parts are used for analysis, and the image is binarized to 0 (black) and 1 (white), as shown in Fig. 5(d).

Step 5: Invert

Invert steps should be conducted on the images to make the autocircle easily and increase the visual condition in Fig. 5(d).

Step 6: Guo–Hall thinning

Noise is present in images after binarization. We used a label to process our images and distinguish between noise and signal. The label is used to determine whether a pixel is connected or disconnected. The same label is used for all connected pixels, and a different label number is provided for each of the connected components. Each connected component is separated, and the characteristics are studied. The processing method of the label is shown in Ref. [R1]. Therefore, the lesion region can be obtained using a label, as shown in Fig. 5(f). The unnecessary noise is filtered, which facilitates the lesion observation.

Step 7: Circle result

According to the lesion position marked in step 6, we determined the central point and boundary of each lesion, defined the position of each lesion, and encircled the lesion. Result is shown in Fig. 5(g).

S3. PCA calculated processes

PCA is a common method in multivariate statistics [40]. This technique has been applied to color technology since 1960. PCA involves the determination of a subspace that is less than the original variable, maintaining of data change in a multivariable data set, and projecting the original data in the subspace for analysis [41]. Principal axis analysis is used to define the principal axis direction of a large number of spectral information and simplify the data. This method calculates the variable with high correlation and independence when the original data are reformed. The principal component is subsequently obtained by analysis. The PCA method can explain the variability of most data [R2]. The principal axis component score is expressed as follows:

$$y_j = a_{j1}(x_{1i} - \overline{x_1}) + a_{j2}(x_{2i} - \overline{x_2}) + \cdots + a_{jn}(x_{ni} - \overline{x_n}), \quad \text{(S16)}$$

where x_{1i} 、$x_{2i} \cdots x_{ni}$ refers to the spectral intensity corresponding to the first, second, and until the nth wave length; $\overline{x_1}$ 、$\overline{x_2} \cdots \overline{x_n}$ refers to the average spectral intensity of the first, second, and nth wavelength; and coefficients a_{j1} 、a_{j2} 、$\cdots a_{jn}$ are the coefficients of the eigenvector when the covariance matrix is determined for each spectrum. According to the Hotelling's rule, the first principal component constitutes the most information in the original data, which can be regarded as the comprehensive index. The information in the second and third principal components in the original data

can be used to classify all groups [R3]. We marked 30 positions as the four kinds of human early esophageal cancerous lesion. Each position includes 400 image elements. The average spectrum of each position is determined for PCA to obtain different eigenvector groups. Six positions from each group of eigenvectors are used as bases to obtain six eigenvalues. After determining the eigenvectors corresponding to two groups of the largest eigenvalue by PCA, Eq. S16 is used to calculate the eigenvalues of each lesion part. Two groups of eigenvalues (a_1 and a_2) are selected to plot the scatter graph. Results are shown in Fig. 6. The a_1/a_2 axis presents the first and second principle components constituting the 97.50% and 1.96% approximation in the lesion's spectrum of white-light endoscopy, respectively. PCA is performed again to determine the distribution using the data of the individual groups. The range is shown in ellipsoid. The equation of the ellipsoid is expressed as follows:

$$\frac{(a_1x+b_1y+c_1)^2}{{d_1}^2}+\frac{(a_2x+b_2y+c_2)^2}{{d_2}^2}=1, \tag{S17}$$

where a_1, b_1, a_2, and b_2 refer to the eigenvector coefficient of the inverse covariance matrix of the groups, the physical meaning of which is axis rotation; and c_1 and c_2 represent the average of the data values of the groups. Groups make a parallel movement of all data points. PCA is conducted so that the center of an ellipse is moved to the original space. The d_1 and d_2 values refer to the feature values of the inverse covariance matrix, which refers to the long axis and half of the short axis of the ellipse.

S4. Sensitivity and specificity of this study

We collected the endoscopic images of patients with different cancer stages to verify the accuracy of triangle region of cancer staging defined in this study: patients 1 and 2 are in intraepithelial papillary capillary loop (IPCL)-V1 squamous cell carcinoma (SCC) stage of esophageal cancer, patients 3 and 4 are in IPCL-V3 SCC stage, patient 4 is also in IPCL-IV severe dysplasia stage, and patient 5 is in IPCL-V1 severe dysplasia stage (Figs. S1–S5). In the five patients, we selected the area (red frame) with even brightness of NBI endoscopic image and converted the image into gray scale. Consequently, image comparison is enhanced. Binarization is carried out to enhance the image. Visual Basic program is adopted to add IPCL pixel coordinates recording NBI endoscopic image after binarization. The recorded pixel coordinates is exported to notepad. NBI endoscopic image is read through the interface of Color Factory program, and the pixel coordinates in the notepad are also read simultaneously. The displaced image and simulation spectrum of the image are reproduced by hyperspectral image technology.

Analysis is performed on lesion area unclear in the endoscopic images of the five patients through hyperspectral image technology to obtain the average spectrum of these unclear lesion areas. The principal component score chart is obtained through PCA (Fig. S6). Most points of patients 4 and 5 are located in the triangle scope of IPCL-

IV severe dysplasia and IPCL-V1 severe dysplasia, respectively. The points of patients 1, 2, and 3 are concentrated in the triangle area of IPCL-V1 SCC and IPCL-V3 SCC. Furthermore, given that the overlap area of patients 1 and 2 is large, which is not good for assessment, we separated these areas and magnified them individually for evaluation (Fig. S6, small figure). We carried out statistics for the evaluation results of the five patients, and results are shown in Table 1. Notably, the possibility of IPCL-V1 SCC and IPCL-V3 SCC stages for patient 1 is 57.6% and 16.2%, respectively. The possibility of IPCL-V1 SCC and IPCL-V3 SCC stages for patient 2 is 48.8% and 21.8%, respectively. In addition, the possibility of IPCL-V1 SCC and IPCL-V3 SCC stages for patient 3 is 8.5% and 68.3%, respectively. The possibility of IPCL-IV severe dysplasia and IPCL-V1 SCC stages for patient 4 is 95.2% and 1.5%, respectively. The possibility of IPCL-V1 severe dysplasia for patient 5 is 93.6%. Therefore, we can rapidly evaluate the possibility of each cancerous stage of patients.

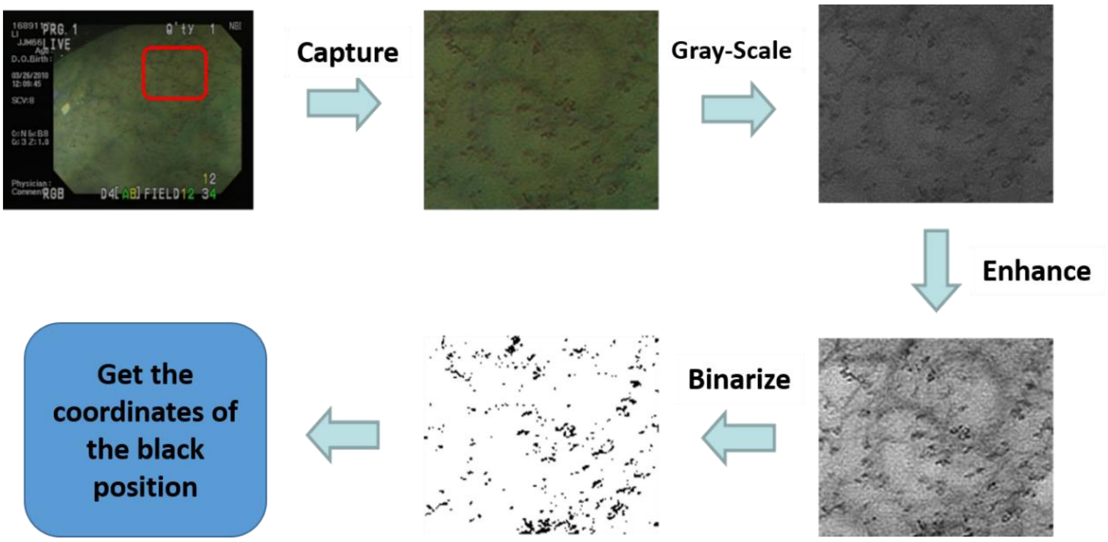

Fig. S1 Image processing of patient 1.

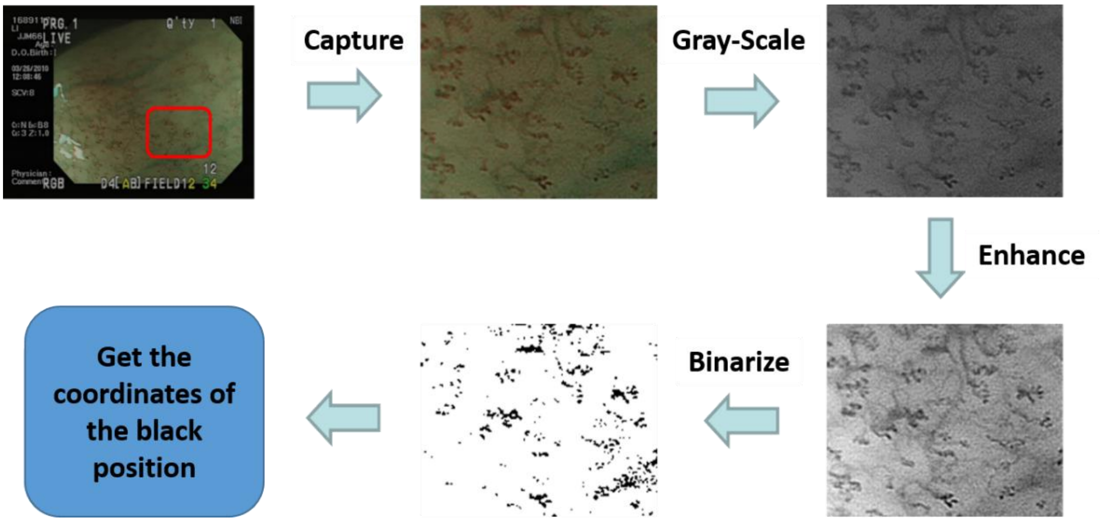

Fig. S2 Image processing of patient 2.

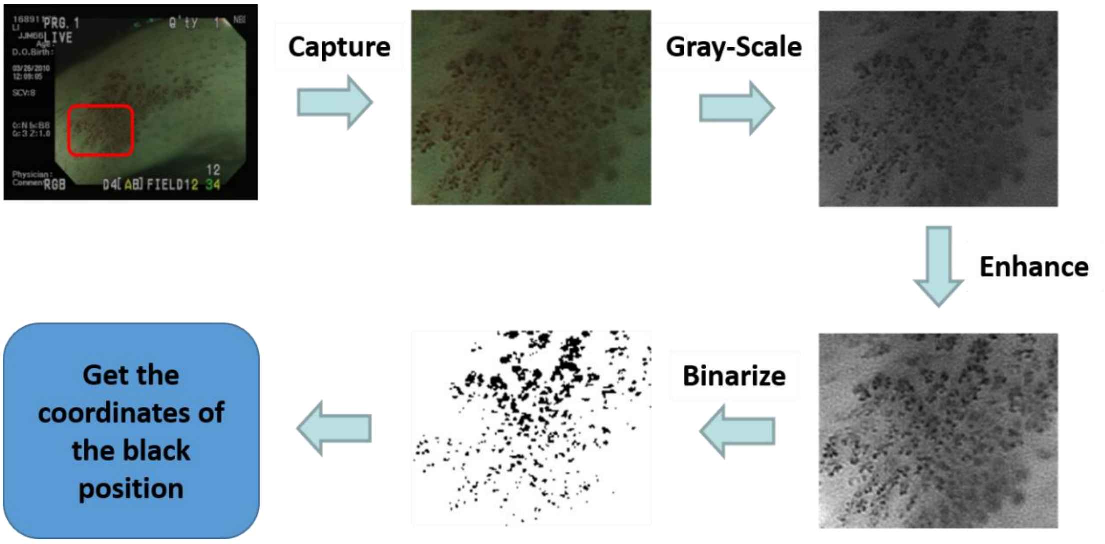

Fig. S3 Image processing of patient 3.

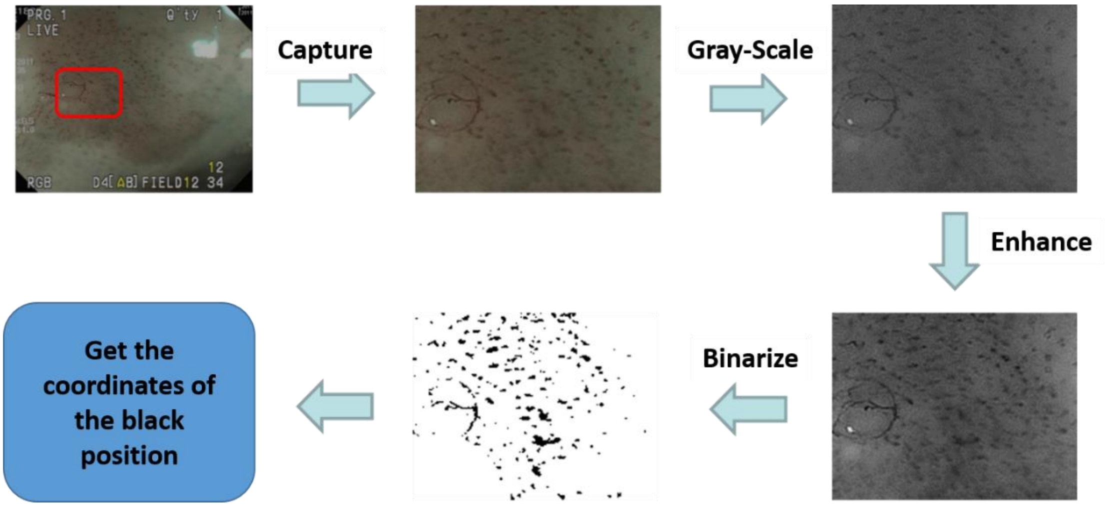

Fig. S4 Image processing of patient 4.

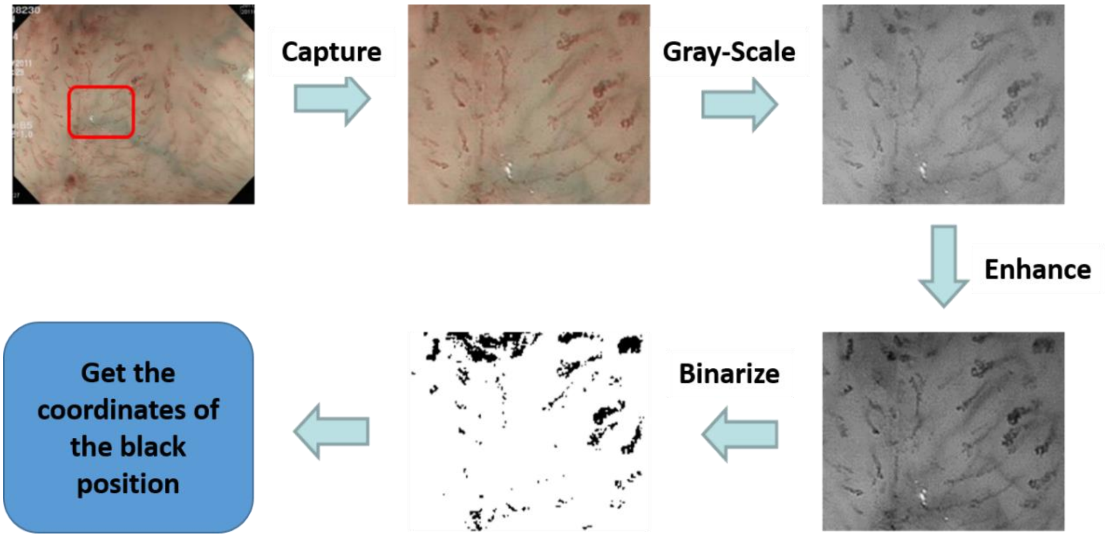

Fig. S5 Image processing of patient 5.

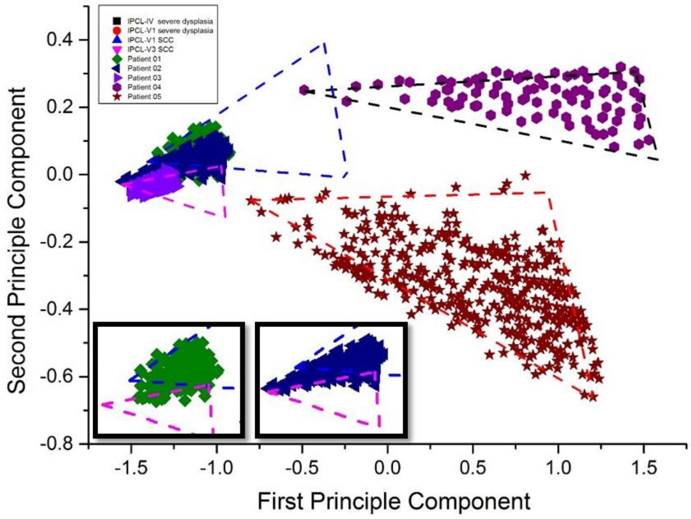

Fig. S6 Principal component distribution diagram of five patients at narrow-band imaging. The two illustrations at bottom left are the enlarged part of patients 2 and 3.

Table S1 Estimated probability of five patients

	IPCL-IV	IPCL-V1	IPCL-V1 SCC	IPCL-V3 SCC
Patient 01	0%	0%	57.6%	16.2%
Patient 02	0%	0%	48.8%	21.8%
Patient 03	0%	0%	8.5%	68.3%
Patient 04	95.2%	0%	1.5%	0%
Patient 05	0%	93.6%	0%	0%

Reference

[R1] D. Phillips, "Image Processing in C: Analyzing and Enhancing Digital Images," R and D Publications, Kanasa, 1994.

[R2] K. Fukunaga, “Introduction to statistical pattern recognition,” San Diego, Academic Press, 1990.

[R3] H. Hotelling, “Analysis of a complex of statistical variables into principal components,” Journal of Educational Psychology 24(6), 417-441, 1933.
